# GIS-ODE: linking dynamic population models with GIS to predict pathogen vector abundance across a country under climate change scenarios

**DOI:** 10.1098/rsif.2024.0004

**Published:** 2024-08-07

**Authors:** A. J. Worton, R. A. Norman, L. Gilbert, R. B. Porter

**Affiliations:** ^1^ Division of Computing Science and Mathematics, University of Stirling, Stirling FK9 4LA, UK; ^2^ School of Biodiversity, One Health and Veterinary Medicine, University of Glasgow, Glasgow G12 8QQ, UK; ^3^ Department of Engineering and Mathematics, Sheffield Hallam University, Sheffield S1 1WB, UK

**Keywords:** GIS, *Ixodes ricinus*, climate change, mathematical modelling, Scotland, ticks

## Abstract

Mechanistic mathematical models such as ordinary differential equations (ODEs) have a long history for their use in describing population dynamics and determining estimates of key parameters that summarize the potential growth or decline of a population over time. More recently, geographic information systems (GIS) have become important tools to provide a visual representation of statistically determined parameters and environmental features over space. Here, we combine these tools to form a ‘GIS-ODE’ approach to generate spatiotemporal maps predicting how projected changes in thermal climate may affect population densities and, uniquely, population dynamics of *Ixodes ricinus*, an important tick vector of several human pathogens. Assuming habitat and host densities are not greatly affected by climate warming, the GIS-ODE model predicted that, even under the lowest projected temperature increase, *I. ricinus* nymph densities could increase by 26–99% in Scotland, depending on the habitat and climate of the location. Our GIS-ODE model provides the vector-borne disease research community with a framework option to produce predictive, spatially explicit risk maps based on a mechanistic understanding of vector and vector-borne disease transmission dynamics.

## 1. Introduction

Species distribution maps and disease risk maps are valuable tools that can inform conservation or disease mitigation strategies, guide future research and are of interest to the public. Risk maps are particularly useful for predicting changes in disease risk owing to certain drivers such as climate change.

The processes to develop such maps involve a diverse range of different types of statistical or mathematical models. Ostfeld & Brunner [1] categorized such models broadly as ‘phenomenological models’ that link current environmental factors and species distributions to predict future distributions and ‘mechanistic models’ that aim to describe the mechanisms by which abiotic factors are thought to drive the demography of the species.

Statistical phenomenological models correlate the focal parameter, such as bird density or disease incidence, with environmental factors such as climate and habitat (as exemplified by Estrada-Pena *et al.* and Ehrman *et al.* [2,3] who assessed environmental correlates of *Ixodes ricinus* tick abundance in Europe). The output algorithm is then linked with geographic information systems (GIS) to visualize variations of the focal parameter over space and time. This approach has been used to produce vector-borne disease risk maps, including for tick-borne encephalitis virus in the Czech Republic [4,5]; *B. burgdorferi* in Bavaria [6] and Trento, Italy [7]; and for *I. ricinus* tick populations in Siebengebirge, Germany [8] and Scotland [9]. These studies primarily use large spatial datasets of vectors, pathogens or disease incidence coupled with spatial environmental data, and can be very useful in describing or predicting vector or disease incidence depending on the variables in each geographic area. However, such statistical models rely on large empirical datasets across wide areas, which are often not available and they lose predictive power when extrapolated beyond the geographical range of the data. Furthermore, such statistical correlations do not provide mechanistic insights into the system and are not inherently dynamic.

In contrast, agent-based models are mechanistic and usually spatially explicit from the outset. Therefore, they are also used for predicting parasite and disease risk across space, such as forecasting changes in Lyme disease risk across Scotland [10] and Europe [11] and SIR-cellular automata models of vector-borne Chagas disease in response to socio-environmental change scenarios [12]. These approaches use more mathematically complex simulations and are more computationally heavy than our approach. Although they are not as data hungry as the statistical models, they often still require data that are not readily accessible for parametrization.

Stokowski & Allen [13] present a novel approach of modelling tick population and infection dynamics that researchers can edit and adapt. It allows users to select transition probabilities for ticks to move between life stages as a next-generation matrix model, and also allows users to choose a duration over which these transitions can occur. Vindenes & Mysterud [14] also use a matrix model to consider the importance of the seasonal questing behaviour of ticks and its interaction with the seasonal behaviour of small hosts. In their model, it is possible to track when in the season a tick fed and whether they moulted in the same year or overwintered as fed, moulting in the following spring/summer. This, in combination with the seasonal variation of small hosts, highlights the importance of small host availability on the overall tick population.

A further modelling approach, recently applied to ticks and tick-borne pathogen spread in eastern North America, used a reaction–diffusion–advection model of partial differential equations and bird movements as the key agent of spatial spread to new areas [15]. Their mathematical model has similar functions and variables to the one we present in this study, but includes diffusion of hosts, since the hosts they are considering are birds and can travel long distances and therefore disperse ticks over a large area. They also superimpose their model onto a map of Africa and include the host suitability of the landscape by considering the proportion of forest cover [15]. While this approach addresses similar questions and includes similar factors to the approach proposed here, it is much more complex mathematically and relies on numerical simulations and more detailed and effective datasets than our approach. In this article, we present a mathematically simpler approach and use ordinary differential equations (ODEs) that are commonly used to predict crucial disease parameters such as the basic reproductive number (*R*_0_) of a pathogen or parasite population density. As ODEs describe the rates of change of populations, they are particularly useful in being inherently dynamic and, being mechanistic, they can help researchers gain mechanistic insights into the system under study. They are often algebraically tractable and this, in particular, can help ODEs provide generalizable biological insights into the system. ODEs on their own are not inherently spatial, but outputs could be meaningfully spatial if linked with GIS, as we demonstrate in this study.

Here we develop a ‘GIS-ODE’ approach, linking dynamic ODE models with GIS to predict variations in tick density over a country (Scotland). We then apply the GIS-ODE model to predict potential changes in tick density in response to projected thermal climate changes for the 2080s. The combined GIS-ODE modelling approach can be readily extended and used in future research, for example, by incorporating vector-borne pathogens to predict disease risk or, more broadly, to understand spatial and temporal dynamics of other species or disease systems.

While several other spatiotemporal modelling approaches have been used to produce predictive risk maps of vectors and disease changes in response to climate change, as mentioned above, this GIS-ODE approach provides a powerful tool that is dynamic and mechanistic, yet mathematically relatively simple so it can be adopted by non-specialists. It can be used to counter some of the shortcomings of approaches that are purely phenomenological. This tool can be applied at a spatial scale appropriate for the population in question or in line with the type of data available and would be applicable in many different scenarios. Here, we focus on tick populations to form a model foundation for future work, which could extend the framework to include tick-borne pathogens. The parameters that drive the ODEs in our study are land use, which is related to the densities of wildlife that are hosts to ticks and temperature, which directly affects tick activity and phenology.

Ticks are important vectors of zoonotic disease-causing pathogens in Europe, transmitting the tick-borne encephalitis complex of viruses, *Anaplasma phagocytophyllum*, *Babesia* and *Rickettsia* species and *Borrelia burgdorferi sensu lato*, the complex of bacteria that cause Lyme disease, among others [16]. *Ixodes ricinus* ticks are of particular health concern because they are ubiquitous across Europe and are generalist feeders, which allows for pathogen transmission among different host species [17]. Alongside climate warming and other environmental and anthropogenic changes, the incidence of several tick-borne diseases, such as Lyme disease, has been increasing in northern Europe (e.g. [18]) and is predicted to increase further in some areas (e.g. [11]). These increases in disease incidence and risk are likely to be partly explained by similar reported increases in vector (*I. ricinus* tick) populations in many parts of northern Europe (reviewed by [19]). Since *I. ricinus* abundance and distribution over Europe are correlated with both climate and habitat [2,3] their reported increases are probably owing to a combination of land use change, increases in hosts (especially deer [20]) and a warming climate [19]. It is, therefore, relevant and timely to produce a predictive risk map of tick densities based on climate, habitat and hosts under current climate and climate change scenarios as a way of demonstrating our combined GIS-ODE approach.

Scotland is an ideal country for pioneering this approach as the issue of ticks and tick-borne disease risk is of increasing concern with reported increases in tick abundance [21–23] and Lyme disease incidence [24]. Furthermore, there are datasets available of *I. ricinus* abundance at a large number of sites throughout Scotland and several relevant studies on *I. ricinus* in the Scottish context (e.g. [25–27]) that can provide locally relevant parameter values and available national-scale GIS-based environmental information.

## 2. Material and methods

Our approach was to develop a general mechanistic dynamic model to predict tick density based on temperature- and host-dependent seasonal tick dynamics (including rates of larval, nymphal and adult tick activity, interstadial mortality and feeding rates). The ODE model was then parametrized with GIS-based spatial environmental information on climate and land cover types as these factors have been shown to greatly influence *I. ricinus* tick distribution and abundance [2,3]. Each land cover type was assigned different host densities and simulations were run for each 3 × 3 km^2^ cell of Scotland to predict (i) tick abundance spatially over Scotland and (ii) how tick abundance may change temporally under low, medium and high climate-warming scenarios.

The general ODE model tracks the change in tick density over time by incorporating the known stages of the tick life cycle, including questing, moulting, reproduction and mortality. Although temperature may affect the rates of development, oviposition and mortality [28] there is a lack of published quantitative data on these effects for *I. ricinus* that could usefully underpin this in a model. However, there are data on the timing of emergence of ticks from overwintering and on tick activity [27–29], so these are both assumed to be temperature dependent in the model (see appendix A for further details). While it is possible that climate warming may also affect hosts, especially their phenology, there is a paucity of published data demonstrating the nature and extent of this in the UK. Therefore, the model assumes that habitats and hosts remain the same under the climate changes modelled.

Host densities vary depending on the habitat type and land management practices in the model. Tick density varies depending on host densities with adult females feeding exclusively on larger hosts (deer, sheep and hares [17,28,30]). The probability of a tick finding a host is assumed to be directly proportional to the density of each host species in a particular area and the number of ticks that find a meal depends on the maximum number of ticks that each host type can carry at each time step. The different cells are parametrized using GIS data on local temperature and habitat, allowing the ODE model to make predictions depending on that local information.

To assess the potential impact of climate-warming scenarios on tick densities the model was run under three warming scenarios (low, medium and high) with linearly increasing yearly temperatures until the 2080s. Low, medium and high temperature increases were based on UK climate projections data [31] and compared to a simulation with no temperature increase.

## 3. Results

**Figure 1.**
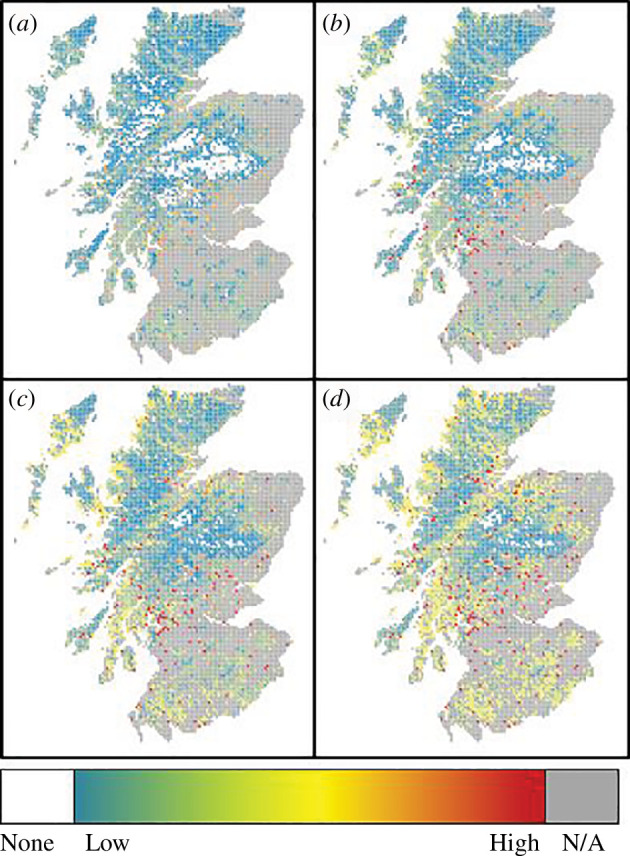
Maps of predicted *I. ricinus* tick densities after 70 years under scenarios of (*a*) no change in temperature, i.e. current climate; (*b*) a final temperature increase of 1°C; (*c*) a final temperature increase of 2.5°C; and (*d*) a final temperature increase of 4°C. Colours represent predicted relative tick densities, with white representing no ticks, cold colours (blue) representing low densities and orange or red representing high predicted tick densities. Grey areas represent areas containing habitats for which there are no robust data on tick abundance, such as arable farmland and urban environments, and which we assume to have negligible tick densities.

For the recent thermal climate, the model predicted fairly low *I. ricinus* tick densities for much of Scotland (coloured blue to green in figure 1*a*), although some small local patches of higher tick densities were predicted, scattered throughout the country (coloured yellow to red in figure 1*a*).

**Figure 2.**
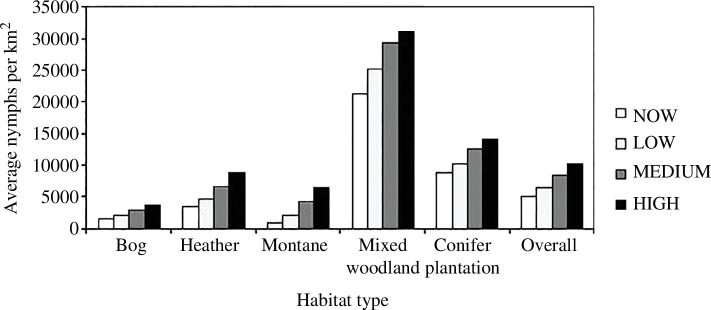
Predicted mean *I. ricinus* nymph densities for each habitat modelled under each climate change scenario (high, medium and low emissions, and no warming representing the current climate). The category ‘heather’ refers to upland moorland and includes rough grassland. ‘Overall’ represents the mean of all habitats modelled.

When no temperature change was included, simulating the recent thermal climate, the model predicted the highest tick densities in mixed woodland habitats (around 22 000 nymphs km^−2^), which is 2.4 times the density of questing nymphs predicted in conifer forest (the habitat with the second highest predicted nymph density) and approximately 30 times the density predicted in montane areas (the habitat with the lowest predicted nymph density; figure 2).

The model predicted an increase in tick densities and a spread in tick distribution over Scotland for all climate-warming scenarios by 2080 (figures 1 and 2). Overall, there was a predicted increase of 26% under the 1°C temperature rise scenario and almost double (99% rise) the average nymph density under the 4°C temperature rise scenario.

The strength of these predicted increases in tick density varied depending on the habitat (figure 2). The largest proportional increases were predicted for montane habitats: many of these areas that were predicted to be tick-free under recent climatic conditions (higher altitudes with cold climates; white areas in figure 1*a*) were predicted to become warm enough to allow sustained tick populations by 2080 (figure 1*b–d* shows a clear reduction in the white (zero-tick) areas over Scotland). By 2080, under the 4°C temperature rise scenario, the model predicted that only the highest peaks will remain too cold for maintaining *I. ricinus* tick populations (figure 1*d*). The model predicted that blanket bog habitats would experience the smallest absolute increases in tick density (figure 2).

## 4. Discussion

The GIS-ODE model presented here integrates a dynamic mechanistic ODE model based on the current understanding of tick biology and parametrized for different habitat types in a GIS to predict *I. ricinus* tick densities over Scotland. By focusing on host habitat suitability, we predicted how areas that were previously unsuitable for tick survival or activity owing to cooler temperatures may become suitable in the future as the climate warms. In addition, the model predicted new potential tick densities for areas where ticks already exist.

The model suggested that, under the recent baseline climate conditions, higher tick densities should be found in lower altitudes and more wooded areas. While we do not expect the quantitative predictions, i.e. the exact values of the tick densities, to be accurate, the qualitative predictions agree with previous studies in Scotland. Higher tick densities have been found in mature woodlands when compared to open grass or heather habitats [32,33] and at lower altitudes [25]. These predictions also reflect documented associations of *I. ricinus* distribution and abundance over Europe with respect to climate and woodland cover [2,3].

Our model predicted that under climate warming all habitats included in the study could experience an increase in tick density and ticks will spread to higher altitudes. Validating what models predict in response to climate change is notoriously challenging without conducting surveys after the climate has changed, such as in 2080. However, although the exact quantitative increases in tick densities are not expected to be accurate, the qualitative patterns are what we might expect from previous empirical studies: Gilbert [25] used altitude as a surrogate for climate change in upland and montane habitats in Scotland and found dramatic increases in *I. ricinus* as the climate warmed (i.e. at progressively lower altitudes). Observed shifts to higher altitudes and/or latitudes over time have also been reported in Norway [34], Sweden [35,36] and Czech Republic [37,38] and Alfredsson *et al.* [39] found unfed *I. ricinus* questing for the first time in Iceland. The authors suggested that increases in average temperatures over the last few decades support conditions which are now favourable for *I. ricinus* ticks. Our GIS-ODE qualitative predictions concur with those of a previous agent-based model which predicted increases in Lyme disease risk in Scottish woodlands and at higher altitudes in response to climate warming [10].

The GIS-ODE model predicted different strengths of effect depending on the habitat. It predicted large proportional increases but small absolute increases in tick densities in montane habitats (in concurrence with predicted Lyme disease risk [10]), only slight increases in blanket bog, while woodland habitats were predicted to experience substantial absolute increases (as per [10]) but small proportional increases in tick densities. The host and temperature basis of the model provided a mechanistic insight into these differences: high altitudes in Scotland are currently beyond the climate envelope of *I. ricinus* [25]. Therefore, when the climate warms enough for these high altitudes to come within the climate envelope of *I. ricinus*, an increase in tick population from zero to low still represents a large proportional increase. It is important to note that for these montane habitats, temperature rather than host availability was the key factor limiting the tick population growth; there were hosts available, such as red deer and mountain hares, but, as suggested by Gilbert [25], the temperatures are too cold for too long to allow ticks to be active for prolonged periods. This means that their likelihood of being encountered by a host is greatly diminished; this is likely to be compounded by slower development and oviposition rates at cooler temperatures [40].

In contrast, blanket bogs exist well within the climate envelope of *I. ricinus*, so an increase in temperature has less of an effect on the proportional increase in ticks than for montane habitats; instead, host availability is likely to be the key factor limiting tick population growth in blanket bogs. For example, Gilbert [26] found dramatically lower *I. ricinus* densities in blanket bog compared to adjacent habitats that shared the same climate, and this was primarily owing to deer preferring to spend time in the other habitats rather than in the blanket bog. Woodlands, on the other hand, were predicted to experience large absolute increases in tick densities. This could be because tick population growth owing to increased temperature is not inhibited by host availability in woodlands. However, the proportional increase in ticks was not very large for woodlands simply because the baseline tick densities were already high for the recent climate. The distinction between temperature- or host-limited tick population growth is an important one highlighted by our model and is crucial for making predictions of future tick and tick-borne disease risk under scenarios of different types of environmental change, such as changes in land use and host availability.

It is important to note that the densities and increases in ticks predicted in this GIS-ODE model are potentials, that is, the model predicts the suitability of the area for *I. ricinus* in terms of temperature and hosts, so if there are ticks present they could reach the specified density. If there are no ticks in that area initially, the model still predicts the potential density they could reach if they were introduced into that area.

In the interests of simplicity, the host densities were assumed to stay constant throughout each simulation. However, this is not often the case in nature; for example, the densities of many hosts, such as rodents, moorland grouse and woodland birds, vary over the season as young ones are produced over the summer, and this is predicted by models to play a role in the seasonal cycle of ticks and tick-borne pathogens [41]. The addition of host seasonality is a possible step to take for future models. Similarly, host densities are treated as uniform across all areas of the same habitat. For example, all woodland areas are assumed in our model to have identical densities of birds, small mammals and deer, but in reality, host (and therefore tick) densities will vary with different woodlands depending on finer scale habitat characteristics [3].

Some hosts may display population cycles between years, as observed in some populations of red grouse and field voles, which would likely contribute to additional variation in tick densities in addition to the longer term climatic effects. In their long-term study, Ostfeld *et al.* [42] found that the drivers for the interannual density of *Ixodes scapularis* nymphs were complex, dependent on host density, climate variation and acorn abundance. The model presented here aims to make long-term, large-scale predictions and, for this purpose, using average values is reasonable, as it allows us to focus on the predicted large differences between broad habitat types and predicted long-term changes caused by climate, with the caveat that there will, of course, be variation within habitats and regions beyond the predictions of the model.

Owing to the lack of quantitative information and for simplicity, we did not allow habitats or hosts to change in response to the Scottish climate-warming projections used in this model. Most of Scotland is well within the climate envelopes of the hosts in the model, so we expect there will be relatively little change, except that perhaps some host species may be able to spend more time at higher altitudes as the climate warms. Although there may be other indirect effects of climate warming on hosts, such as food availability, phenology and parasite burdens, the lack of information on these potential factors prevents us from including them in the current model.

A further detail that could be incorporated for future GIS-ODE models includes tick generation times in response to climate warming. In particular, a mechanism could be introduced which allows ticks to feed, develop to the next stage and then feed again within the same year for warmer climates where interstadial development times are faster [43]. Temperature may also affect tick fecundity and survival, but research is needed to produce reliable parameter estimates for inclusion in models. In addition, there is evidence that *I. ricinus* adapt their activity to their local climate, such that ticks in warmer climates (such as central France or southern England) are much less active at cooler temperatures than are ticks from cooler climates (such as Scotland [27]). The Gilbert *et al.* [27] study suggests that, if ticks in Scotland eventually adapt to the projected warmer climate, they may no longer be so active at cool temperatures. Instead, they may be active only at warm temperatures, as currently exhibited by French *I. ricinus*. Incorporating this climate change adaptation into the model would require adjustments to the questing function to dampen or delay the currently predicted increase in activity with increased temperature [27]. Incorporating climate adaptation into our model would be expected to buffer the predicted increase in tick densities and distribution, so the predicted tick increases will probably be less than suggested here.

A current limitation of this method is that it is not possible to allow movement between cells. This is unlikely to be important in our case study given the scale of the cells in the GIS and the hosts we are considering, but if there are hosts that travel over large areas or if climate change made some areas more suitable/attractive for hosts (and hence movement between cells becomes more likely) then that might be a factor which would need to be included. To achieve this, mechanistic movement models, similar to those used by Tardy *et al.* [15], could be used to explore the direct movement of ticks via hosts.

The model presented here combined a dynamic tick population model with GIS information to visualize potential tick densities at a national scale. However, tick densities may or may not relate to tick-borne disease risk, depending on the pathogen in question, the relative densities of competent and incompetent transmission hosts, as well as tick density. Therefore, future models need to introduce mechanisms that describe the transmission of pathogens between vector and host, which will allow predictions to be made for disease risk across Scotland. This would be done by adapting the models from, for example, Gilbert *et al.* [44]. These already include the hosts and therefore habitats so temperature dependence would be added and the ODE models integrated into the GIS.

More generally, this method can be readily adapted to model the dynamics of other vector systems, such as mosquitoes and midges without substantial alterations. The model provides a baseline for future work incorporating pathogen dynamics, such as tick-borne, midge-borne or mosquito-borne pathogens such as louping-ill, bluetongue or West Nile virus. The model was run here for Scotland but is easily applicable to other areas and could be run for any region with data on minimum and maximum temperatures, habitat type and host communities. This GIS-ODE modelling approach could be adapted to any other vector system in any other geographic region provided that reasonable estimates are available on key vector life history parameters and how the vector activity and/or mortality responds to temperature. In addition, information is needed on environmental (habitat and/or climate) preferences for the main hosts of the vector, as is appropriate GIS environmental data that link to habitats or hosts relevant to the vector. An advantage of this methodology is that it allows us to understand the interaction between long-term changes in temperature and the subsequent predicted dynamic change in focal species populations on a different time scale and in response to those changes. The granularity of the model predictions depends on the scale of the environmental GIS data. In our case, we classified the grid cell types according to temperature and land cover and assumed the density of host types and then ran the ODE model on each cell. The method is not mathematically complex but could be computationally expensive depending on the number of grid cells and cell classifications, which will vary depending on the ecological system and the problem being considered. Although we have made some simplifying assumptions in this case study, more complexity could be included if the data were available. For example, temperature-dependent host population dynamics and seasonality could be incorporated.

Although we have chosen here to visualize the outputs on the GIS at a particular timepoint, it would be possible to show interim values and indeed to have an animation that shows the dynamics of the changes over time.

The predictions of the model are informative in alerting us to which broadscale habitat types and areas may be most affected by climate change (such as higher altitudes) and which areas (such as blanket bog) are likely to be more resilient, owing to the tick populations being limited by other factors such as hosts. This may aid tick control strategies or public awareness campaigns of tick-borne disease risk.

## 5. Conclusion

While this case study model makes a number of simplifying assumptions, it has allowed us to identify which geographic areas and habitats might be particularly vulnerable to increased tick densities owing to climate warming. Interestingly, using a mechanistic approach based on tick biology has also allowed us to distinguish which areas have tick densities that are likely to be limited by climate and which may be limited by host availability. While we developed the GIS-ODE approach to predict tick densities over Scotland, this approach could be easily used for other areas and other vector species, and pathogens could be added to the model, enabling predictions of disease risk. Indeed, this methodology could be used more broadly to understand the dynamic response of populations over time to a variety of environmental changes, and provides a neat new method in the modelling toolbox for researchers to choose from.

## Figures and Tables

**Figure 3.**
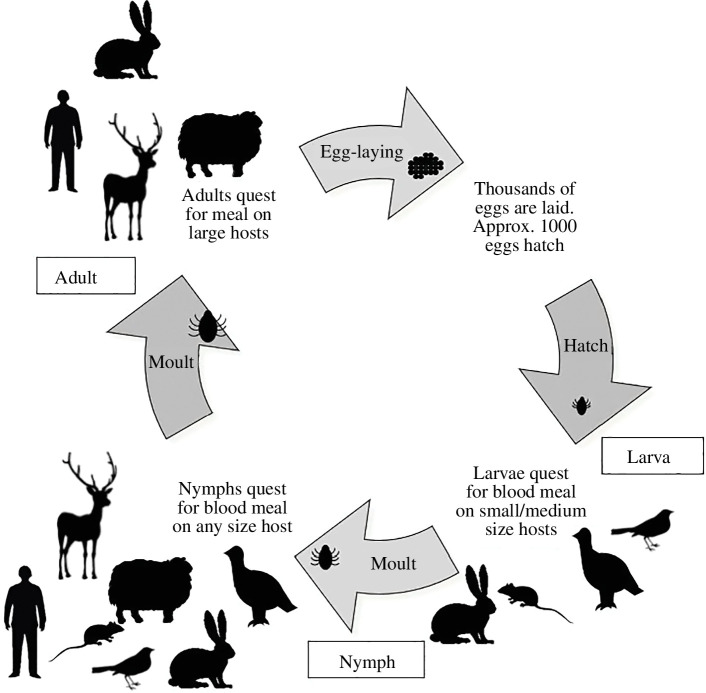
Schematic diagram of the *I. ricinus* life cycle with examples of the types of hosts that each instar tends to feed on.

**Figure 4.**
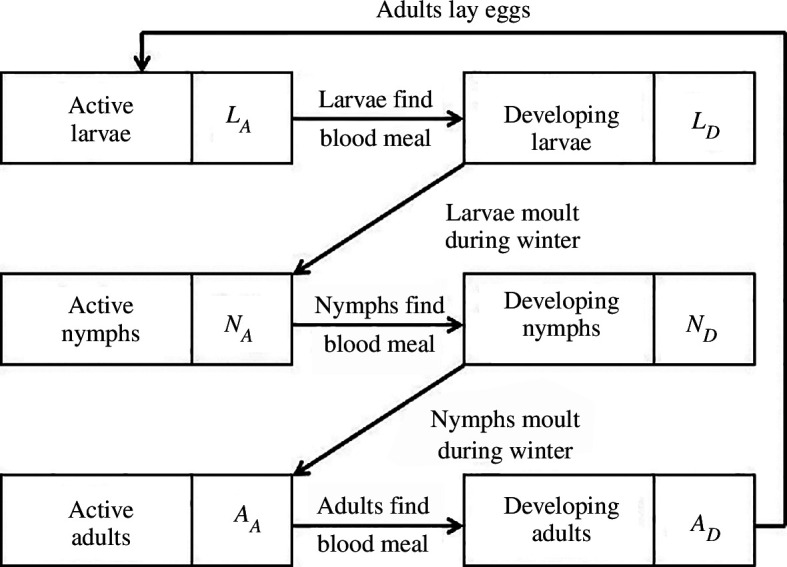
Schematic outline of the conceptual model describing the *I. ricinus* life stages and key activities (feeding, moulting, active, developing) used in the model.

**Figure 5.**
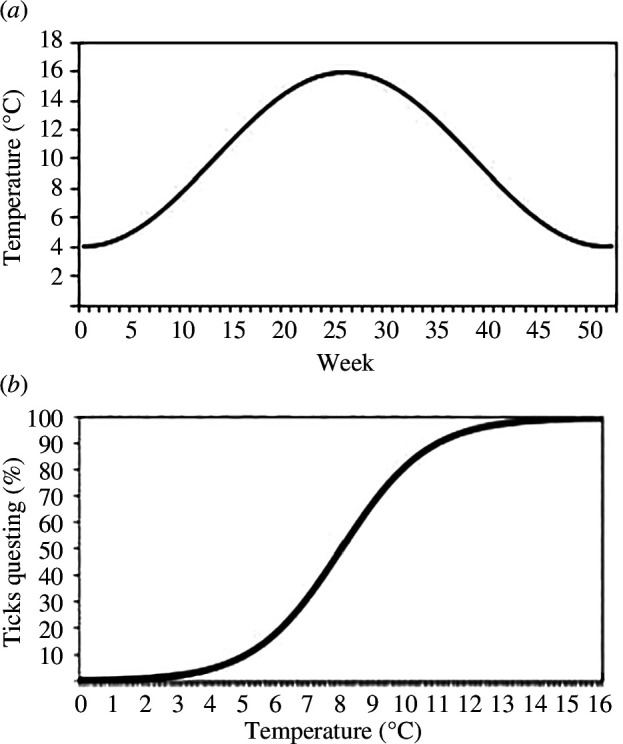
(*a*) Temperature variation across the year for baseline temperature. (*b*) Percentage of active ticks assumed to be questing, P(t) , for temperatures between 0 and 16°C. The function is based on empirical data from [27].

**Figure 6.**
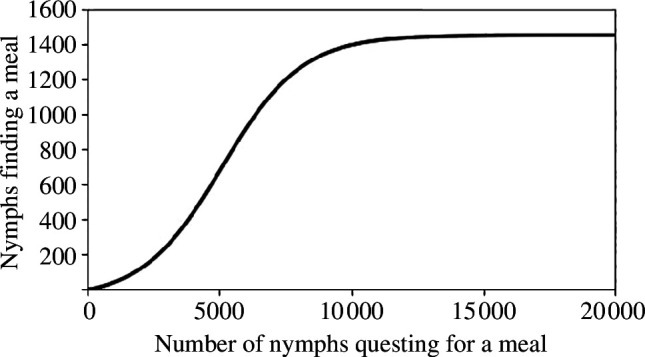
An example of the *I. ricinus* feeding function S(x,K) , where x is the number of ticks per km^2^ questing and K is the weekly feeding limit, set to 1500.

## Data Availability

The Mathematica code which ran the model on the different cells and the Excel spreadsheet which includes the data for each cell type which was used to visualize the results in the GIS are available at datastorre.stir.ac.uk entitled ‘GIS data ticks in Scotland plus model code’. Supplementary material is available online [45].

## Appendix A: Model description

### A.1. ODE aspect of the approach

The model consists of a series of differential equations that run throughout the year, followed by a discrete time step to move onto the next year. This time step is taken at the time when temperatures are at their coldest, and therefore when tick activity would be at its lowest. The initial conditions for year n+1 are taken from the end of the preceding year, with a proportion lost over the winter, as discussed below.

The *I. ricinus* life cycle (figure 3) develops from the egg, through two immature stages (larva and nymph) to the adult stage and usually takes around three years to complete. Each immature stage requires a single blood meal, taking a few days, from a suitable vertebrate host before developing to the next stage and the adult female requires a blood meal before producing eggs. Because ixodid ticks spend the vast majority of their life away from the host in vegetation or the ground, their activity and survival are highly influenced by the surrounding local environment. The activity of *I. ricinus*, like most invertebrates, is inhibited by low temperatures [27] and they also tend not to seek hosts (quest) at low humidities or high saturation deficits in order to avoid desiccation [43]. Adult female *I. ricinus* feed primarily on large mammals such as deer, sheep or hares while the immature stages can also feed on smaller vertebrates such as mice, voles and birds (e.g. [30]). For forecasting future tick activity or abundance, these factors must therefore be taken into account.

The tick life cycle in our model is divided into three active life stages (larvae, nymph and adult). Each life stage is split further into one group which has yet to feed that year (which we term ‘active’ to represent ticks that are available to quest if the conditions are appropriate) and one group that has already fed (which no longer quest and we term here ‘developing’) (figure 4). While ticks feeding twice (i.e. as a larva and then as a nymph or as a nymph and then as an adult) within one season has been documented [43], these observations were made south of Scotland in warmer climates where temperature-dependent development times are faster. It is likely that the colder summers in Scotland would result in only a very small proportion of ticks being able to feed, develop to the next stage and then feed for a second time in the same season. While including the ability for an individual tick to feed twice per year if temperatures were warmer would be realistic to some extent, it is complicated by the fact that we know that ticks can adapt their development to local temperatures [27] so there is no clear definition of what temperature that would occur at. Therefore, for simplicity, in the model presented here, all ticks are assumed to feed once per year.

The time period used in the model is weekly, as this fits with the typical period spent by ticks feeding on a blood host.

#### A.1.1. Feeding

For ticks of any given stage to find a blood meal, Randolph [46] identified four key aspects of tick development between stages: entering the stage (i.e. development from the previous stage), questing for a meal, successfully feeding on a host and mortality. These have all been incorporated into the model presented here.

As tick questing activity is generally very low during winter in Scotland, a temperature boundary (*T*_1_) is set in the model, below which all active ticks are assumed to be not questing. A second temperature boundary (*T*_2_) is set, above which all active ticks are available to quest if conditions are right. Between the two temperature boundaries, we assumed a linear progression as ticks gradually become available to quest. Field studies have observed that ticks generally emerge after winter when average weekly temperatures reach at least 7°C [47]. We assume there will be individual variation around this mean such that some ticks will emerge below and some will emerge above this average; therefore, for the model, we assume that there is variation in individual emergence around a mean of 7°C, and so *T*_1_ is set to 5°C and *T*_2_ is set to 9°C.

The equation for the function which describes the pattern of emergence from overwintering between these two temperatures is given below:

$$F\left(t\right)=\left\{ \begin{array}{ll} 0, & T\left(t\right)\leq T_{1},\\ \frac{T\left(t\right)-T_{1}}{T_{2}-T_{1}}, & T_{1}<T\left(t\right)<T_{2},\\ 1, & T\left(t\right)\geq T_{2}, \end{array}\right.$$

where $T\left(t\right)$ is the average temperature for the current week (figure 5*a*).

Note that ticks which have emerged from the winter are not necessarily questing at every time point, as questing activity is limited by factors such as temperature [48]. Therefore, we introduce a questing function $P\left(t\right)$ to the model, which describes ticks within the active state questing for a host. This questing function is based on data from experiments conducted by Gilbert *et al.* [27], who determined the proportion of potentially active, emerged ticks which were questing at different temperatures. For ticks collected in Scotland, they found that most ticks began questing between 6°C and 12°C, with almost 100% questing at 15°C. The experiment was conducted at high relative humidity (approx. 90%), which is fairly applicable to natural conditions on a forest floor in a wet temperate country such as Scotland. The following equation approximates the results from [27]:

$$P\left(t\right)={\left(1+exp\left[0.75\left(8-T\left(t\right)\right)\right]\right)}^{-1}.$$

A graph of this function for temperatures between 0 and 16°C can be seen in figure 5*b*.

The probability of a tick finding a host is assumed to be directly proportional to the density of each host species present at a particular site. It is also assumed that the number of ticks that find a meal in a given time period increases with the number of ticks that are questing up to a threshold limit that depends on how many hosts are available. Therefore, the upper limit for the density of ticks that can feed in a given week has to be estimated. This weekly feeding limit is calculated by using the available data to estimate the maximum number of ticks each host type can carry at each time step and multiplying that by the density of each host species present (table 1). The following density-dependent relationship is then used to model the density of ticks which find a host in a given week:

$$S\left(x,\;K\right)=\frac{GK}{G+\left(K-G\right)\exp\left(rx\right)}-G,$$

**Table 1.** Values used in the model for maximum ticks feeding per individual host each week on each host species and densities (per km^2^) of each host species in each habitat type included within the model. Tick burden information from which these values were estimated is given in the footnotes.

parameter	passerine birds	red grouse	mountain hare	red deer	roe deer	small mammals
*tick burdens on each host species*						
larva	3.11^a^	15^c^	63.7^f^	15.18^c^	10.78^i^	1.61^k^
nymph	0.25^a^	4^c^	37.4^f^	70.41^c^	23.9^i^	0.03^k^
adult	0^a^	0^c^	11.4^f^	81.25^c^	29.82^i^	0^k^
*density of each host species in each habitat (per km^2^*)						
blanket bog	0	0	8^c^	1^g^	0	0
upland moorland	0	0, 50, 100^d^	8^c^	0, 5, 15^h^	0	0
montane	0	20^e^	16^c^	0.1^g^	0	0
mixed woodland	70	0	0	0^g^	4^j^	8500
conifer plantation	35^b^	0	0	0^g^	3^j^	4250^b^

^a^ [49].

^b^ Densities of hosts in conifer plantation are assumed to be half of that of semi-natural woodlands (L. Gilbert, unpublished data).

^c^ L. Gilbert, unpublished data.

^d^ None, medium and high scenarios of grouse density, as explained in the text. High scenario value chosen as is a typical density of grouse moors.

^e^ Ptarmigan density, which in the absence of official population data [53], is estimated to be lower than typical grouse densities.

^f^ K. M. Laurenson, unpublished data.

^g^ [52].

^h^ None, medium and high deer density scenarios of deer density, as explained in the text. Based on values taken from averaged Deer Management Group counts up to 2006.

^i^ [50].

^j^ [54].

^k^ [51].

where x is the number of ticks of a given stage which are questing, *G* and r are constants which determine the shape of the curve and K is the weekly feeding limit discussed above. This is an adaptation of the solution of the logistic equation and satisfies the assumptions discussed above.

There are no available data to parametrize this function further but we estimate that in order for 95% of questing ticks to find a meal, 8K ticks per km^2^ need to be questing. Therefore, we set G=50 and $r=\frac{\ln\frac{G}{19\left(K-G\right)}}{8K}.$

An example of this function is shown in figure 6.

Mortality rates for ticks that have not yet fed are based on values given in [55], scaled to reflect weekly rather than daily mortality. All ticks that have not fed by the time temperatures drop below $T_{1}$ are assumed to have died, as Gregoryev [56] demonstrated that even in optimal conditions of high humidity 100% of unfed larvae and nymphs died within 3 months. All adults are removed from the model at the end of each year, assumed to have reached the end of their lifespan.

#### A.1.2. Interstadial development

As ticks that have successfully fed are assumed not to feed a second time that year, all fed ticks stay in the developing class until the end of the year, at which point they progress to the active class of the next instar. As this class is equivalent to the behavioural diapause category in the paper presented by Dobson *et al*. [55], we use their relevant mortality rates. Over winter, mortality is introduced to the model during the discrete step from one year to the next, such that the final number of ticks in each developing stage is reduced by the relevant proportion to become the initial condition for the next stage in the following year.

#### A.1.3. Birth

As larvae will have hatched by the start of the tick activity season in early spring, all births within the model occur at the start of each year before ticks are active. The model developed by Gilbert *et al.* [44] assumed the number of eggs laid by each adult female to be 1400 and Buczek *et al.* [57] record hatching success as 89.6%, giving an estimate of 628 larvae produced from each adult which has successfully fed, assuming a 1:1 adult sex ratio.

#### A.1.4. Model equations

The model for the tick activity season is, therefore, given by the following set of coupled nonlinear differential equations:

,$$\frac{dL_{A}}{dt}=-S\left[F\left(t\right)\times P\left(t\right)\times L_{A}\left(t\right),L_{host}\right]-b_{L}L_{A}\left(t\right)$$

,$$\frac{dL_{D}}{dt}=S\left[F\left(t\right)\times P\left(t\right)\times L_{A}\left(t\right),L_{host}\right]$$

,$$\frac{dN_{A}}{dt}=-S\left[F\left(t\right)\times P\left(t\right)\times N_{A}\left(t\right),\;N_{host}\right]-b_{N}N_{A}\left(t\right)$$

,$$\frac{dN_{D}}{dt}=S\left[F\left(t\right)\times P\left(t\right)\times N_{A}\left(t\right),\;N_{host}\right]$$

,$$\frac{dA_{A}}{dt}=-S\left[F\left(t\right)\times P\left(t\right)\times A_{A}\left(t\right),\;A_{host}\right]-b_{A}A_{A}\left(t\right)$$

$$\frac{dA_{D}}{dt}=S\left[F\left(t\right)\times P\left(t\right)\times A_{A}\left(t\right),\;A_{host}\right],$$

where L are larvae, N are nymphs and A are adult *I. ricinus* ticks and the subscripts *A* and *D* refer to the ‘active’ and ‘developing’ states of each tick stage. The parameter definitions, values used and the source of those values are given in table 2.

**Table 2.** Definitions of model parameters, numerical values used and source of values. All values are per week.

parameter	value	explanation and justification
a	628	average successful birth rate per adult tick [44] and [57]
$b_{L}$	0.047	natural mortality rate for each tick stage’s active state [55]
$b_{N}$	0.024	
$b_{A}$	0.009	
$b_{WL}$	0.00995	natural mortality rate for larvae developing into nymphs during winter [55]
$b_{WN}$	0.00333	natural mortality rate for nymphs developing into adults during winter [55]
$L_{host}$	varied	weekly feeding limit (maximum number of ticks feeding on each host) for each tick stage and each host. Varies for different tick stages, hosts and therefore habitat types (see table 1)
$N_{host}$	varied	
$A_{host}$	varied	
$T_{1}$	5	temperature (°C) at which ticks begin to emerge from overwintering [58]
$T_{2}$	9	temperature (°C) at which all ticks have emerged from overwintering [58]

### A.2. GIS aspect of the approach

#### A.2.1. Spatial variation in the model

Using ArcGIS 10 (Environmental Systems Research Institute, 2014), all spatial environmental data we used over Scotland were split into a 3 × 3 km^2^ grid, with each cell containing the data for the maximum and minimum annual temperature based on Met Office long term data (1971–2000), as well as the habitat type. We categorized habitat as: blanket bog, upland moorland/rough grassland, montane, mixed woodland or conifer plantation. Built-up areas and prime arable land were not considered in the model as these areas are considered to generally have extremely low tick densities (L.G., unpublished data) or owing to a lack of information associating tick abundance to environmental factors in these areas. The uplands of Scotland are highly managed landscapes, so host densities are a direct result of the management objectives of the land holding unit. Of particular importance to our model is the fact that some areas of upland moorland are managed specifically to maximize densities of red grouse since they are valuable game birds. Red grouse managers often maintain very low deer densities in order to keep tick numbers low [59]. Conversely, other areas of upland moorland aim to maintain high deer densities for commercial red deer hunting, and these areas therefore tend to have few red grouse. Other upland areas of Scotland are less specialized and maintain a mix of hunted species. Therefore, upland moorland cells in the model were categorized into three simplified host community groups: (i) high grouse densities, no deer; (ii) medium grouse densities, medium deer densities; (iii) no grouse, high deer densities.

The cells were categorized by temperature and habitat types into 34 groups, chosen so that each group covered approximately 100 cells. The details of these groups can be seen in the electronic supplementary material.

For each of these groups, a simulation was run on Mathematica v. 9 [60] using appropriate host densities for that habitat (see table 1). It should be noted that we chose to use Mathematica but any method of running the simulations could be combined with the GIS in this way. A sine function was used to represent temperature over each year, where the maximum and minimum temperatures relate to the values for each 3 × 3 km^2^ cell.

#### A.2.2. Predicting changes in tick densities with climate change

Using these values of temperature, host densities and habitat categories, simulations were run for 70 years, while we increased temperatures linearly over this time period in order to represent UK Climate Projections data [31]. As the average annual temperature in Scotland is estimated to increase by 1–4°C by the 2080s, depending on the emission scenario, the models were run under three climate change scenarios: low, medium and high temperature increase, with rises of 1°C, 2.5°C and 4°C, respectively. These outputs were compared with simulations that were run with no change in temperature to represent the current climate.
